# Brain Activation of Negative Feedback in Rule Acquisition Revealed in a Segmented Wisconsin Card Sorting Test

**DOI:** 10.1371/journal.pone.0140731

**Published:** 2015-10-15

**Authors:** Jing Wang, Bihua Cao, Xueli Cai, Heming Gao, Fuhong Li

**Affiliations:** 1 Brain and Cognitive Neuroscience Research Center, Liaoning Normal University, Dalian, 116029, China; 2 School of Psychology, Jiangxi Normal University, Nanchang, 330022, China; 3 Psychological Research and Counseling Center, Southwest Jiaotong University, Chengdu, 611756, China; 4 School of Psychology, Liaoning Normal University, Dalian, 116029, China; University Zurich, SWITZERLAND

## Abstract

The present study is to investigate the brain activation associated with the informative value of negative feedback in rule acquisition. In each trial of a segmented Wisconsin Card Sorting Test, participants were provided with three reference cards and one target card, and were asked to match one of three reference cards to the target card based on a classification rule. Participants received feedback after each match. Participants would acquire the rule after one negative feedback (1-NF condition) or two successive negative feedbacks (2-NF condition). The functional magnetic resonance imaging (fMRI) results indicated that lateral prefrontal-to-parietal cortices were more active in the 2-NF condition than in the 1-NF condition. The activation in the right lateral prefrontal cortex and left posterior parietal cortex increased gradually with the amount of negative feedback. These results demonstrate that the informative value of negative feedback in rule acquisition might be modulated by the lateral prefronto-parietal loop.

## Introduction

The Wisconsin Card Sorting Test (WCST) is one of the most widely used neurocognitive measures to evaluate cognitive flexibility, problem-solving, rule learning, as well as brain disorders [1–12]. During the task, participants need to match test cards to reference cards according to three possible rules: color, number, or shape. No instructions for how to match the cards are given but feedback is provided after each match, which helps participants to obtain the correct classification rules. Initially unaware of the correct rule, participants sort the cards randomly or formulate a hypothesis for sorting and test it by trial and error. After some successive correct sorting, the rule will be made invalid by negative feedback (NF), which informs participants that what was formerly right is now wrong, and they are required to search for a new rule.

The feedback has two main values: valence and informative value [13, 14]. The former specifies whether the current behavior is right or wrong. The latter indicates that what information the feedback provided for us. For example, when participants received negative feedback they need to shift rules by discarding the invalid rule and searching for a new rule [15, 16]. Studies on the valence of NF have suggested that the medial prefrontal cortex (PFC), including the anterior cingulate cortex (ACC), is active following NF [17–19]. NF also activates the regions previously related to cognitive control and response selection, including the dorsolateral prefrontal cortex (DLPFC) and the dorsal anterior cingulate cortex (dACC) [20–22]. Studies on the informative value of NF have found that the bilateral orbital frontal, ventrolateral prefrontal cortex (VLPFC, area 47/12), caudate nucleus and the inferior frontal sulci, which are parts of the dorsal Brodmann's areas (BA) 45/44, play an important role in set-shifting [23–27]. For example, Monchi et al. [12] used the WCST to compare the brain activation of NF to that of positive feedback. They revealed that a cortical basal ganglia loop was more active during the reception of NF compared with the positive feedback. They proposed that a cortical basal ganglia loop including the VLPFC was involved in the process of rule shifting that was guided by the NF.

However, Monchi et al. [12] did not differentiate the NF in rule acquisition from that in rule shifting. Specifically, the function of NF in rule acquisition is to exclude a possible hypothesis and guide participants to search for other hypotheses. However, the main function of NF in rule shifting is to discard the classification rule that was valid for previous successive trials and search for a new classification rule. To our knowledge, only a few investigators have attempted to isolate the informative value from valence of NF [16, 27]. No fMRI study has explored the brain regions associated with the NF in rule acquisition.

We developed a segmented WCST to study the brain activation associated with NF in rule acquisition. The stimulus was similar to the standard version of the WCST. In each trial, one target card and three reference cards were displayed. Each reference card shared only one perceptual attribute (e.g., shape, color, or number) with the target card. Participants were required to choose a reference card that was the same class with the target card. Feedback was presented after each match. Before the task, participants were informed that the classification rule might pertain to one of three perceptual attributes of stimulus. If participants received NF at the first match, the excluded rule at the first match was not allowed to choose at the second and third matches. Otherwise, no feedback was displayed on the screen. As a result, they would acquire the rule after two attempts at most and they must match correctly at the third attempt. Accordingly, participants’ rule acquisition behavior can be classified into three conditions. In the 0-NF condition, participants luckily acquired the rule at the first match, so they did not receive any NF in this condition. In the 1-NF condition, participants failed at the first match, but responded correctly at the second and third match, so they receive one NF in this condition. In the 2-NF condition, participants failed at the first and second match, but responded correctly at the third match, so they receive two NFs in this condition.

The difference between the 2-NF condition and 1-NF condition is the amount of NF. We compared the difference in brain activation evoked by the 2-NF condition with that of the 1-NF condition. Because the crucial function of NF in rule acquisition is to guide the participants to rule out the invalid hypothesis and turn to searching for a new hypothesis [19, 25, 27], and previous studies demonstrated that the LPFC is associated with the rule search process [28–33]. Therefore, we predicted that the LPFC might be more active in 2-NF condition than in 1-NF condition.

## Materials and Methods

### Participants

Sixteen healthy right-handed volunteers (nine male, seven female; age range: 19–26 years; mean age: 21 years) participated in this experiment. All participants met the criteria for magnetic resonance imaging (MRI) scanning (i.e., had no metallic implants, no history of claustrophobia, and a head size compatible with the custom head coil). All participants reported normal or corrected-to-normal vision and the absence of neurological or psychiatric impairments. This study has been approved by the Institutional Review Board at Liaoning Normal University. We had obtained appropriate ethics committee approval for the research reported, and all subjects gave written informed consent in our experiment.

### Experimental task

**Fig 1:**
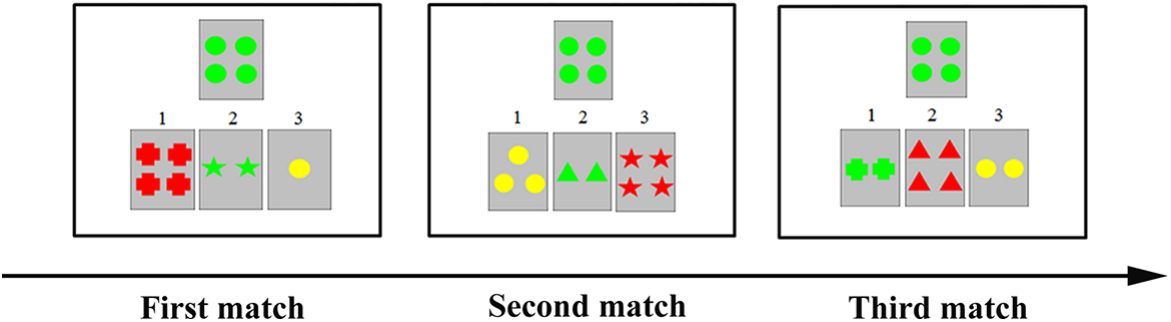
Sample materials used in a trial. The stimulus presented on each card had three attributes including shape (cross, circle, triangle, and star), number of the shapes (1, 2, 3, and 4), and color of the shapes (red, green, yellow, and blue). Each reference card shared one attribute with the target card.

A segmented WCST modified from the standard version of the WCST task was used [6, 7]. One target card was presented in the middle of the higher visual field, and three reference cards appeared under the target card (Fig 1). On each trial, the target card was fixed, but the reference cards were changed. Participants were required to match the target card to one of the three reference cards on the basis of the shared perceptual attribute (e.g., color, shape, or number).

**Fig 2:**
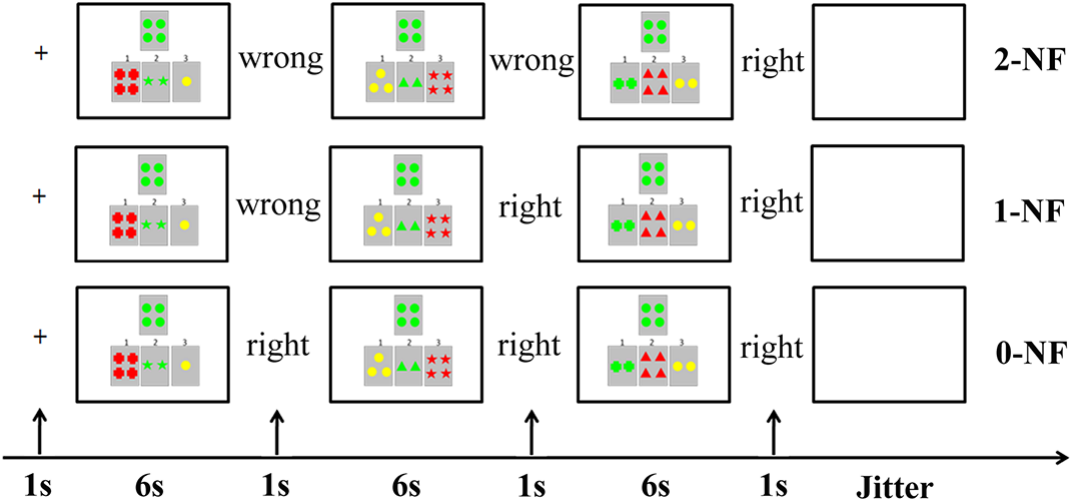
Three conditions and the experimental procedure of a trial. First, participants viewed a fixation cross, followed by a stimulus display. The stimulus required a left index, left middle finger, or right index finger response within the time (6000ms) that the stimulus was displayed. The stimulus display was followed by the feedback for 1000 ms. A pseudorandom jitter of 2000 ms, 4000 ms, or 6000 ms was added at the end of a trial.

Each trial started with a fixation cross. A new target card was then presented on the first screen. The participant chose one of the three reference cards by pressing a button for 1, 2, or 3. The length of each match period depended on the participant’s response time, but the allowed maximum response time was 6000 ms. If the participant did not responded within 6000 ms, “No response” would appear on the screen. After the response, the feedback of was displayed, remaining on the screen for 1000 ms. Then, the target and the new reference cards would appear on the second and third screens, and participants would respond with new match. The rule was constant for one trial, and was changed between trials (Fig 2). Before the experiment, the experimenters explained the procedure to the participants in details, and participants were required to perform a practice session outside of the scanner.

Depending on the participants’ response and feedback, three conditions were defined. In the 2-NF condition, participants received two NFs. In the 1-NF condition, participants received a NF after the first match. At the second and third match, they responded correctly. In the 0-NF condition, they luckily gave the correct responses for all of the three matches. There were five runs.Each run had 3 conditions and 18 trials. Each condition had 6 trials in one run. A total of 90 trials were equally assigned to the three conditions, which were executed by the E-prime software. Because each condition has the same number of trials in each run, the possibility of participants receiving negative feedback and positive feedback is 2/3 and 1/3, respectively, at the first occasion. If participants received negative feedback at the first occasion, the possibility of receiving negative feedback and positive feedback at the second occasion is 1/2 and 1/2, respectively. If participants received positive feedback at the first occasion, the possibility of receiving negative feedback and positive feedback at the second occasion is 0 and 1, respectively.

### Image acquisition

Scanning was performed with a standard whole-head coil using a 3.0 T full-body magnetic resonance imaging scanner (Siemens, Erlangen, Germany). The blood oxygenation level-dependent (BOLD) contrast was acquired using echo planar T2*-weighted imaging. The parameters used were: repetition time = 2000 ms, echo time = 30 ms, field of view = 220 × 220 mm, flip angle = 90°, matrix size 64×64, 32slices, slice thickness = 3mm (0.99 mm gap), in-plane resolution = 3.43 × 3.43 mm, 246 volumes per run. The stimulus was presented using a magnet-compatible projector that back-projected visual images onto a screen mounted above the participant's head. The experimental task was programmed using E-Prime package (version 2.0; Psychology Software Tools, Inc., Pittsburgh, PA; hwww.pstnet.com). Responses were obtained using a magnet-compatible response system.

### Data analysis

Image preprocessing and analysis were performed using SPM8 (Welcome Department of Cognitive Neurology, London, UK). The first two volumes in each session were excluded. Images were slice-time corrected, motion corrected, re-sampled to 3 × 3 × 3 mm isotropic voxels, normalized to the Montreal Neurological Institute (MNI) space, and spatially smoothed using an 8 mm full width at half maximum isotropic Gaussian kernel. After preprocessing, participants whose head motion exceeded 3.0 mm and 3.0° were excluded from the study. At the first level, the time series in each voxel was high-pass filtered to 1/128 Hz and modeled for temporal autocorrelation across scans with an AR (1) model. The first level (individual level) statistical analysis was conducted with the general linear model to form statistical parametric maps of the Paired T-statistic. The contrasts were as follows: 2-NF vs 1-NF, 2-NF vs 0-NF, and 1-NF vs 0-NF. Individual contrast images were entered into a second level analysis to make inferences at the group level. In order to assess areas associated with NF function (i.e., negative vs positive), a conjunction analysis (2-NF vs 0-NF ∩ 1-NF vs 0-NF) was conducted. A FDR corrected of *p <* 0.05 and a cluster size of *k >* 20 was used to search for significant regions of conjunction at the whole brain level.

ROI analyses were performed using the Marsbar toolbox in SPM8 [34]. The definition of the ROIs was based on Monchi al et. [12]. We used the peak coordinates as the center of ROIs, which were all 6mm radius sphere ROIs. Following this, the BOLD responses (beta values) of each condition were extracted.

## Results

### Whole-brain analysis

**Fig 3:**
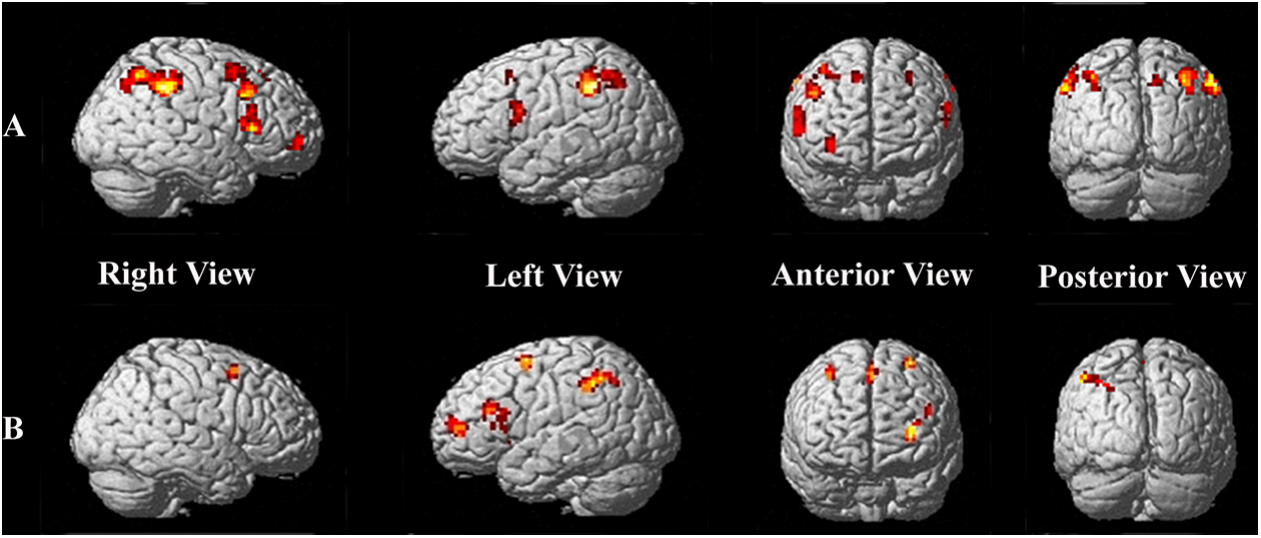
Brain activation on 2-NF vs 1-NF and conjunction analysis. A is the areas where were more active in the 2-NF condition than in the 1-NF condition. B is the active areas of conjunction analysis (2-NF vs. 0-NF ∩ 1-NF vs. 0-NF)**.**

In comparison with the 1-NF condition, the 2-NF condition activated the bilateral inferior frontal Gyrus (IFG), bilateral DLPFC, bilateral posterior parietal cortex (PPC), and right putamen (Table 1 and Fig 3). Activation in the bilateral IFG and bilateral PPC reached significance at a high threshold (FDR corrected, *p* < 0.05), while the right putamen reached a lower level of significance (uncorrected, *p*< 0.001).

**Table 1:** Regions more active for 2-NF condition than 1-NF condition.

	BA	X	Y	Z	T scores	Voxels
**R Inferior Frontal Gyrus**	**44,45**	**48**	**18**	**9**	**5.07**	**76**
**L Inferior Frontal Gyrus**	**44**	**-57**	**12**	**15**	**4.75**	**36**
**R Middle Frontal Gyrus**	**9,10**	**48**	**15**	**39**	**4.99**	**119**
**L Middle Frontal Gyrus**	**8**	**-27**	**12**	**45**	**4.66**	**22**
**R Superior Frontal Gyrus**	**32**	**15**	**24**	**45**	**5.19**	**23**
**R SupraMarginal Gyrus**	**40**	**51**	**-36**	**42**	**5.79**	**156**
**L Inferior Parietal Lobule**	**40,7**	**-54**	**-39**	**45**	**5.7**	**121**
**R Angular Gyrus**	**7**	**42**	**-69**	**42**	**4.85**	**63**
**R Superior Parietal Lobule**	**7**	**36**	**-60**	**51**	**5.15**	**92**
**R Precuneus**	**7**	**15**	**-66**	**45**	**6.58**	**57**
**R Putamen**	**47**	**30**	**21**	**-3**	**4.77**	**31** **

L, left; R, right; BA: Brodmann’s areas; x, y, z, coordinates of the centroid of the region in MNI coordinates.

**p < 0.001, uncorrected

Compared with the 0-NF condition, the 2-NF condition activated the left supplementary motor area (SMA), bilateral PFC, bilateral PPC, left inferior temporal Gyrus, right fusiform Gyrus, left lingual gyrus, bilateral calcarine fissure, right caudate body, left thalamus, and bilateral declive. In comparison with the 0-NF condition, the 1-NF condition activated the right SMA, left PFC, bilateral PPC, left middle occipital gyrus, right lingual gyrus, right insula, left caudate body, and left declive. To further reveal the differences between the two NF conditions and 0-NF condition, we performed a conjunction analysis [35] of 2-NF vs 0-NF ∩ 1-NF vs 0-NF. As is shown in Table 2 and Fig 3, the left SMA, bilateral PFC, left PPC, bilateral insula, and left lateral globus pallidus were activated.

**Table 2:** Regions more active for 2-NF vs 0-NF ∩ 1-NF vs 0-NF.

	BA	X	Y	Z	T scores	Voxels
**L Supplementary Motor Area**	**6**	**-6**	**18**	**48**	**5**	**128**
**L Inferior Frontal Gyrus**	**13**	**-39**	**21**	**12**	**4.23**	**146**
**R Middle Frontal Gyrus**	**6,46**	**33**	**9**	**54**	**3.89**	**20**
**L Middle Frontal Gyrus**	**6,10**	**-30**	**51**	**6**	**4.01**	**45**
**L Inferior Parietal Lobule**	**40**	**-45**	**-39**	**42**	**4.12**	**127**
**L Insula**		**-27**	**24**	**6**	**4**	**46**
**R Insula**	**47**	**33**	**18**	**0**	**3.76**	**23**
**L Lateral Globus Pallidus**		**-21**	**-12**	**0**	**3.54**	**26** **

L, left; R, right; BA: Brodmann’s areas; x, y, z, coordinates of the centroid of the region in MNI coordinates.

**p <0.001, uncorrected

### ROI analyses

**Fig 4:**
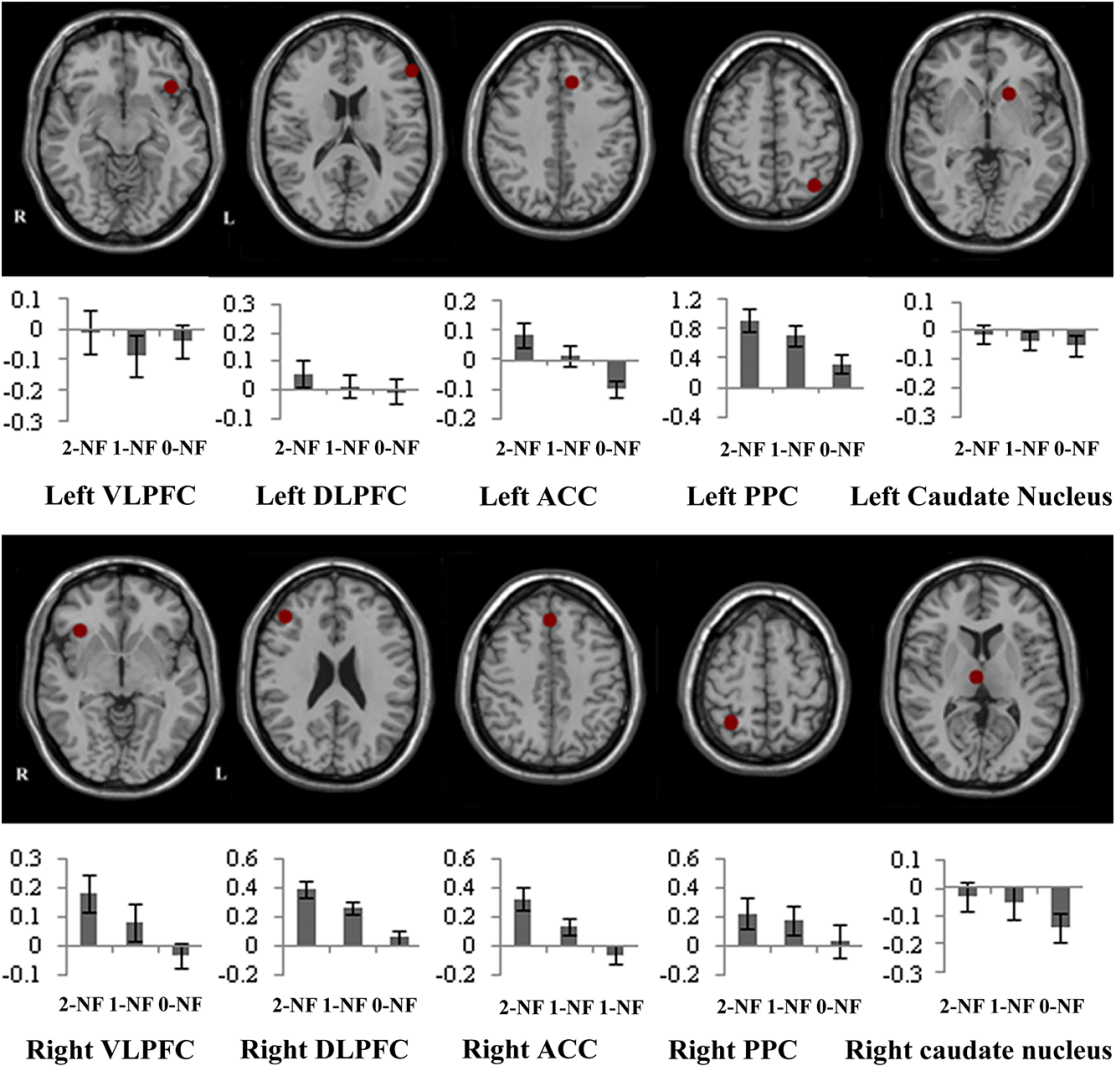
ROIs and the results. Percent signal change for each condition was selected from the ROIs. Error bars reflect standard deviation of the mean.

The ROI analyses were performed for ten defined regions, which were selected based on Monchi al et. [12]. Two of the ROIs were in the DLPFC [(-55, 36, 17), (44, 36, 24)]; two were in the VLPFC [(-40, 23, -10), (36, 25, -4)]; two were in the ACC [(-10, 27, 40), (2, 34, 44)]; two were in the caudate nucleus [(-18, 17, -2), (5, -15, 8)]; two were in the PPC [(-34, -62, 55), (30, -53, 59)]. Because the coordinates in Monchi’s study were the Talairach coordinates [36], we changed them into MNI coordinates [37].For each ROI, beta values were extracted from each participant and submitted to repeated measures analysis of variance (Fig 4)**.**

The analysis for the right VLPFC revealed a main effect of condition, *F*(2, 30) = 11.4, *p* < 0.01. Pairwise comparisons with Bonferroni correction demonstrated that more activation was observed in the 2-NF condition than in the 1-NF condition (*p<* 0.05). The activation in the right VLPFC was also larger in the 1-NF than in the 0-NF condition (*p*< 0.05). For the left VLPFC, a main effect of condition was not found.

The analysis for the right DLPFC revealed a main effect of condition, *F*(2, 30) = 15.3, *p* < 0.001.Pairwise comparisons with Bonferroni correction revealed that more activation was observed in the 2-NF condition than in the 1-NF condition (*p* < 0.05). The activation in the right DLPFC was also larger in the 1-NF condition than in the 0-NF condition (*p* < 0.01). For the left DLPFC, a main effect of condition was not found.

The analysis for the right ACC revealed a main effect of condition, *F*(2, 30) = 10.8, *p* < 0.01. Pairwise comparisons with Bonferroni correction revealed that more activation was seen in the 2-NF condition than in the 1-NF condition (*p* < 0.05). The activation in the right ACC was also larger in the 1-NF condition than in the 0-NF condition (*p* <0.05). The analysis for the left ACC revealed a main effect of condition, *F*(2, 30) = 10.7, *p* < 0.005. Pairwise comparisons with Bonferroni correction indicated that there was more activation in the 1-NF condition than in the 0-NF condition (*p* <0.05).

The analysis for the left PPC revealed a main effect of condition, *F*(2, 30) = 13.3, *p* < 0.01. Pairwise comparisons with Bonferroni correction demonstrated that more activation was seen in the 2-NF condition than in the 1-NF condition (*p* < 0.05). The activation in the left PPC was also larger in the 1-NF condition than in the 0-NF condition (*p*< 0.05). For the right PPC, a main effect of condition did not reach statistical significance. The analysis for the bilateral caudate nucleus did not reach the significance in the main effect.

## Discussion

Previous studies have investigated the cognitive function and related brain activation of NF in the WCST. However, the functions of NF in the different stages of the WCST were not distinguished from each other [12, 27]. We designed a segmented WCST based on the traditional WCST.Our task required the participants to encode, store, and update the common attribute among the stimulus and feedback. Participants would depend on the working memory to complete the segmented WCST. However, this task was not a simple working memory task. Instead, it had the same core cognitive processing as the WCST, which was to abstract the common attribute between the reference card and target card and obtain the right classification rule depending on the feedback. In this task, rule shifting was not involved and we directly examined brain activation in response to NF in rule acquisition. According to the participants’ responses and feedback, there were three types of experimental conditions: the 2-NF, 1-NF, and 0-NF conditions.

The three conditions differ in the amount of NF. Previous studies have demonstrated that feedback has two values in a rule-learning task: valence and informative value [13, 14]. In rule shifting, the valence function of NF plays a more important role. However, in rule acquisition, the informative value of NF might play a crucial role [20, 38]. In rule acquisition, each NF informed participants that the previous hypothesis was invalid and a new hypothesis should be advanced. Compared with the 1-NF condition, the 2-NF condition evoked more activation in the LPFC reflecting that the informative value of NF was more intensively embodied.

The VLPFC has been demonstrated to be active when participants receive NF [18, 39]. In rule learning, the informative value of NF is to rule out the wrong or invalid hypothesis. A study has shown that the VLPFC can help people to keep unwanted memories out of mind [40]. In the 1-NF condition, participants had to rule out one invalid hypothesis before they found the right classification rule. In the 2-NF condition, participants had to rule out two invalid hypotheses before they found the right classification rule. The activation of the right VLPFC was larger in the 2-NF condition than in the 1-NF condition and 0-NF condition, indicating that the VLPFC, especially the right VLPFC, was involved in the process of ruling out the invalid hypothesis. When the amount of hypothesis exclusion increased, the activation of the right VLPFC increased linearly.

The activation of the DLPFC, especially the right DLPFC, increased with the amount of NF. Previous studies have revealed that the DLPFC is involved in rule searching, rule selection, and information updating [19, 41–44]. For example, Crescentini, Seyed-Allaei [29] demonstrated that the mid-DLPFC played an important role when participants found the rule from the stimulus and generated an appropriate hypothesis. Thus, the DLPFC may play an important role to integrate the related information to generate an appropriate hypothesis in the category learning task.

In the WCST, NF activated the PFC-basal ganglia circuits [12]. The activation of basal ganglia was also observed in our study. Compared with the 1-NF condition, the 2-NF condition activated the right putamen, when a less strict threshold was applied. Compared with the 0-NF condition, the 2-NF and 1-NF conditions activated the right caudate nucleus and left caudate nucleus when a less strict threshold was used. These results reveal that the basal ganglia and LPFC might play a role in the informative value of NF in rule acquisition.

In addition, compared with the 0-NF condition, the 1-NF and 2-NF conditions activated not only the LPFC, but also the left SMA. The SMA was possibly recruited in searching for the new hypothesis, being primarily responsible for preparing a rule-based action response. That is, the SMA might form a new connection between the stimulus perceptual attribute of the stimulus and a rule-based response [45, 46].

Finally, we found that compared with the 0-NF condition, the NF conditions activated the bilateral insula. Previous studies indicated that the insula is related to negative events [47, 48]. The activation of the insula might be associated with the slight negative emotion evoked by the NF conditions (i.e. failure in finding the rule) in the present task. However, the activation of the insula was not reported in Monchi’ study. It is possible that, in Monchi’s study, there were many positive feedbacks preceding the NF. The positive emotion caused by successive positive feedback might weaken the negative emotion evoked by the NFs. On the contrary, there was no positive feedback preceding the NF in our study. Particularly in the 2-NF condition, participants made two successive wrong match responses, which possibly evoked negative emotion. Accordingly, it can be speculated that the activation of the insula is not related to the informative value of NF, but to the emotional experience evoked by the valence of NF.

In conclusion, a segmented WCST was used to study the brain activation associated with the informative value of NF in rule acquisition. The activation of the LPFC including the VLPFC and DLPFC may reflect the informative value of NF. The activation of the insula may reflect the valence of NF related to the emotional experience that occurred after the failure of rule acquisition. These results expand our understanding about the different functions of NF in rule learning.
